# Parallels of Resistance between Angiogenesis and Lymphangiogenesis Inhibition in Cancer Therapy

**DOI:** 10.3390/cells9030762

**Published:** 2020-03-20

**Authors:** Dennis Jones

**Affiliations:** Department of Pathology and Laboratory Medicine, Boston University School of Medicine, 670 Albany Street, Boston, MA 02118, USA; djones1@bu.edu; Tel.: +617-358-2751

**Keywords:** lymphangiogenesis, angiogenesis, treatment resistance, metastasis

## Abstract

Metastasis is the primary cause of cancer-related mortality. Cancer cells primarily metastasize via blood and lymphatic vessels to colonize lymph nodes and distant organs, leading to worse prognosis. Thus, strategies to limit blood and lymphatic spread of cancer have been a focal point of cancer research for several decades. Resistance to FDA-approved anti-angiogenic therapies designed to limit blood vessel growth has emerged as a significant clinical challenge. However, there are no FDA-approved drugs that target tumor lymphangiogenesis, despite the consequences of metastasis through the lymphatic system. This review highlights several of the key resistance mechanisms to anti-angiogenic therapy and potential challenges facing anti-lymphangiogenic therapy. Blood and lymphatic vessels are more than just conduits for nutrient, fluid, and cancer cell transport. Recent studies have elucidated how these vasculatures often regulate immune responses. Vessels that are abnormal or compromised by tumor cells can lead to immunosuppression. Therapies designed to improve lymphatic vessel function while limiting metastasis may represent a viable approach to enhance immunotherapy and limit cancer progression.

## 1. Introduction

Angiogenesis, the development of new blood vessels, is a feature of many solid cancers [1]. Recruitment of blood vessels is critical to support tumor growth past 1–2 mm in diameter [2]. Vascular endothelial growth factor-A (VEGF-A) is the most comprehensively studied and perhaps potent mediator of sprouting angiogenesis. Through binding to VEGFR-2 vascular endothelial growth factor receptor-2 (VEGFR-2)/human kinase insert domain receptor, VEGF-A triggers activation of VEGFR-2 and intracellular signaling mediators that promote endothelial cell proliferation, migration, and survival, as well as vascular permeability and ultimately neovascularization [3]. New blood vessels not only deliver nutrients and oxygen to growing tumors but also provide a route of cancer cell exit to distant organs [4]. Many preclinical models using anti-angiogenesis therapies blocking VEGF-A signaling have broadly prohibited or slowed tumor growth and reduced metastatic spread. There are over 20 drugs with anti-angiogenic activity approved by the FDA for cancer indications [5], with Avastin—humanized anti-VEGF-A monoclonal antibody—being the first granted approval in 2004. Hundreds of clinical trials for multiple solid cancers targeting VEGF-A alone or in combination with other therapies have been initiated. However, despite the growing list of FDA approvals, anti-angiogenesis drugs have had a modest impact on patient survival. For example, Avastin only adds a 4–5 month survival benefit in patients with advanced colorectal cancer [6]. Such discrepancies between mouse and human provide the impetus for understanding the molecular and cellular resistance mechanisms of anti-angiogenic therapy.

Lymphangiogenesis also occurs in many preclinical cancer models and in some human cancers mainly through the production of vascular endothelial growth factor-C (VEGF-C) and VEGF-D, which signal through VEGFR-2 and VEGFR-3 and drive lymphatic endothelial cell (LEC) proliferation, migration, and survival [3]. Unlike inhibiting angiogenesis, blunting lymphangiogenesis—the formation of new lymphatic vessels—has mixed results on primary tumor growth in preclinical models [7,8,9], mainly indirectly due to modulation of the anti-tumor immune response. Several drugs that inhibit VEGFR-3 have been used for cancer indications [10] and overlap exists between the targets of anti-angiogenesis drugs and molecules on lymphatic vessels [11]. Recently, an early stage clinical trial targeting VEGFR-3 was completed, but showed minimal efficacy against tumor growth [12]. However, lymphatic vessels provide a major route for cancer cell dissemination. The expression of VEGF-C and VEGF-D correlates with increased metastasis, invasion, and poor prognosis in several types of cancer [13], in part due to tumor-associated lymphangiogenesis. After invading initial lymphatic capillaries, cancer cells migrate through collecting lymphatic vessels and enter regional lymph nodes, where they form secondary tumors. A fraction of nodal metastases can then exit lymph nodes and spread to distant sites [14,15]. The majority of cancer patients die from distant metastasis [16] and thus treating metastatic disease remains a challenging clinical problem. Since most cancer cells need to access the primary or secondary tumor vasculature to metastasize, inhibiting the growth of tumor-associated vasculature remains an attractive therapeutic strategy.

Bergers and Hanahan [17] describe how tumor cells adapt to anti-angiogenic treatment or are intrinsically resistant to such therapy. Over the past decade, additional experimental data have shed light on anti-angiogenesis resistance mechanisms. Here, we review evidence that suggests targeting tumor-associated lymphatic vessels poses similar challenges as targeting tumor blood vessels.

## 2. Timing

Primary tumor lymphangiogenesis is an early event in cancer progression and may present a narrow window of therapeutic intervention. Unfortunately, about a third of breast, colorectal, and lung cancer patients are lymph node positive at diagnosis [18,19], missing an opportunity to block initial lymphangiogenesis in these patients. In addition to the primary site, lymphangiogenesis also occurs in metastatic organs. In many preclinical studies, lymphangiogenesis can occur in regional lymph nodes and distant organs before metastases have occurred and is thought to create a “lymphovascular niche” that creates a favorable environment for disseminated cells [20]. VEGFR-3 reporter mice revealed lymphangiogenesis in lymph nodes, liver, lungs, and spleens of tumor-bearing mice [21] before metastatic spread. High lymphatic density and lymphatic invasion in metastatic lungs was associated with poor outcome of melanoma patients [22]. Blocking pro-lymphangiogenic VEGF-C signaling after cancer cell colonization of tumor-draining lymph nodes prevented further spread to lungs [23] and inhibiting lymphangiogenesis in distant organs may prevent further dissemination of metastatic cells. However, validated biomarkers predictive of lymphangiogenesis are lacking.

## 3. Target

VEGF-A mediates traditional sprouting angiogenesis, while VEGF-C is a critical regulator of lymphangiogenesis. However, over 40 molecules other than VEGF have been shown to play a role in blood vessel growth [24], illustrating the complexity of inhibiting angiogenesis where alternative angiogenic pathways exist. Likewise, the formation of postnatal lymphatic vessels is not limited to VEGF-C dependent mechanisms. Many factors other than VEGF-C have been shown to stimulate lymphangiogenesis. These include other growth factors, cytokines, hormones, proteins, and peptides, many of which induce lymphangiogenesis independently of VEGFR-3 signaling [25,26]. Together, these data suggest that neutralizing VEGF-C alone may lead to drug resistance facilitated by alternative pro-lymphangiogenic molecules.

In addition to multiple growth factor pathways that are activated and promote vascular sprouting in the tumor microenvironment, molecular crosstalk exists between the blood and lymphatic endothelium. VEGFR-2, a key transducer of angiogenic signaling, is expressed on LECs as well as blood endothelial cells. Likewise, VEGFR-3 is expressed on lymphatic endothelium and also on the endothelium of tumor blood vessels [27]. In a reversal of traditional functions, VEGF-A can promote lymphangiogenesis while VEGF-C stimulates tumor angiogenesis [28]. Neutralization of VEGF-A signaling reduced lymphangiogenesis in models of inflammation and tumor growth [3,29,30]. It is unclear whether histological samples from patients treated with anti-angiogenic therapy have been comprehensively evaluated to assess an effect on tumor-associated lymphatic vessels.

## 4. Metastasis Independent of Lymphatic Endothelial Cell Sprouting

One mechanism that accounts for tumor resistance to anti-angiogenic therapy is blood vessel co-option, where tumors utilize pre-existing blood vessels from the surrounding tissue for nutrients. Highly vascularized tissues such as lymph nodes, where up to 10% of the organ volume is blood vessels [31], are thought to be conducive to blood vessel co-option by tumors [32]. En route to co-opting local vessels, some cancer cells exhibit a replacement pattern [33]; in this case cancer cells replace normal tissue but spare blood vessels. Peritumoral lymphatic vessel density is often significantly higher than intratumoral lymphatic vessel density [34]. In fact, intratumoral lymphatic vessels are often absent [35], suggesting that cancer growth can be destructive to intratumoral lymphatic vessels. Although lymphangiogenesis is found in pre-metastatic tumor draining lymph nodes, we measured a reduction in lymphatic vessel density within metastatic lymph nodes relative to pre-metastatic nodes [32].

**Figure 1:**
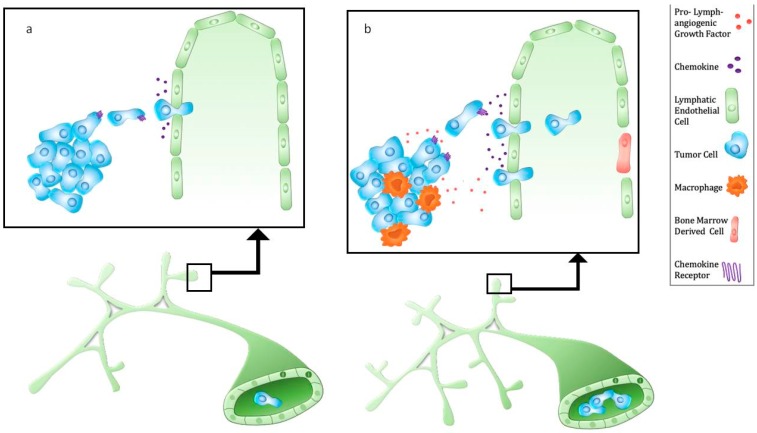
Metastasis via tumor-associated lymphatic vessels. (**a**) Tumor cells express chemokine receptors (e.g., CXCR4, CCR7) which bind chemokines produced by lymphatic endothelium (e.g., CXCL12, CCL21). Lymphatic vessel-derived CCL21 attracts cancer cells that can enter initial lymphatic vessels through interendothelial cell gaps. (**b**) Cancer cells and tumor-associated macrophages secrete pro-lymphangiogenic factors such as VEGF-C, leading to an increase initial lymphatic vessel density and collecting lymphatic vessel diameter. In addition, VEGF-C upregulates CCL21 and increases lymphatic vessel permeability, resulting in enhanced lymphatic metastasis. Of note, bone marrow derived cells can closely associate with or incorporate into lymphatic vessels.

Lymphatic vessel density around the primary tumor and lymphatic vessel invasion are predictors of lymph node metastasis and poor prognosis [36]. However, tumor lymphangiogenesis may be cancer and subtype-specific [7]. Proliferating tumor-associated lymphatic vessels were identified in human squamous cell carcinoma and melanoma [37,38]. In contrast, despite human data and numerous preclinical models that show lymphangiogenesis promotes lymphatic metastasis, analyses of human breast, prostate, and esophageal cancer tissue suggest that cancer cells can invade existing lymphatic vessels (Figure 1) rather than induce proliferation [39,40,41,42,43]. The prognostic significance of lymphangiogenesis in triple negative breast cancer is unclear, yet lymphatic invasion is associated with poor prognosis [44]. Thus, there is still debate as to whether lymphangiogenesis is a pre-requisite for lymphatic metastasis. Tumors that develop in tissues with a dense lymphatic vascular network may exploit pre-existing lymphatic vessels for invasion and metastasis.

Other mechanisms of tumor blood vessel vascularization include intussusception, endothelial progenitor cell-mediated vasculogenesis, vascular mimicry by cancer cells, and differentiation of cancer stem cells into endothelial cells [1,45]. Similarly, lymphatic beds may consist of LECs derived from heterogeneous sources. Recent findings [46] show that the level of circulating myeloid derived lymphatic endothelial progenitor cells, as determined by co-staining of myeloid and LEC markers, strongly correlate with lymphatic metastasis in breast cancer patients. These progenitor cells were shown to incorporate into lymphatic vessels in human and mouse tumors (Figure 1). While there is debate concerning the extent of the contribution of bone marrow derived cells to lymphangiogenesis, these recent studies add to existing data showing that certain populations of bone marrow cells express LEC markers and can function as putative LEC progenitors in pathologic settings such as corneal and kidney transplantation, wound injury, and tumor progression [47,48,49,50,51]. 

## 5. Cancer Cells Use Existing Migratory Cues

A physiological function of lymphatic vessels is to provide chemical signals, or chemokines, for immune cell migration. In addition, many cancer cells overexpress chemokine receptors [52] that allow them to migrate toward lymphatic capillaries, employing the same chemoattraction mechanism used by dendritic cells and T cells. An example is high expression of CCR7 found in various human cancer cells [53,54,55]. C–C chemokine ligand 21 (CCL21), the ligand for CCR7, is constitutively expressed by lymphatic vessels and in secondary lymphoid organs [56] and can be upregulated in response to increased lymphatic flow and inflammatory stimuli [57]. Likewise, lymphatic vessel derived CXCL12 in physiological systems directs dendritic cell migration to lymph nodes [58]. Lymphatic vessels can attract CXCR4-expressing tumor cells to promote lymphatic metastasis [59], and CXCR4 expression is also associated with lymphatic metastasis [60]. Lymphangiogenic signaling can enhance chemokine-driven metastasis, but it appears that tumor cells can independently “hijack” host mechanisms of leukocyte trafficking to facilitate metastasis (Figure 1).

## 6. Myeloid Cell Recruitment

Tumor-associated macrophages (TAMs) are an abundant population in preclinical cancer models and human tumors [61]. Pro-angiogenic monocytes recruited from the bone marrow promote tumor vascularization, growth, and cause resistance to therapy [17]. TAMs are also associated with high lymphatic vessel density and lymphatic metastasis (Figure 1). Macrophages may contribute directly to the lymphatic vasculature through transdifferentiation [49], progenitor differentiation [46], and vascular mimicry [62,63] or indirectly by secretion of pro-lymphangiogenic factors [64]. VEGF-C acts as a chemotactic factor for VEGFR-3 expressing macrophages [65]. In response to paclitaxel, VEGFR-3^+^ macrophages were recruited to breast and lung tumors and mediated chemotherapy resistance by enhancing VEGF-C production that stimulated lymphangiogenesis [66]. Surprisingly, an anti-VEGFR-3 antibody alone did not reduce tumor volume, VEGF-C expression, or the number of lymphatic vessels in the absence of paclitaxel, suggesting that recruited macrophages mediate the lymphangiogenic response to paclitaxel.

In breast cancer models, podoplanin-expressing TAMs promote metastasis [67] by remodeling the extracellular matrix, leading to the release of VEGF-C and VEGF-D to direct lymphangiogenesis. Recently, Evans et al. identified a population of Beta-4 integrin expressing TAMs that promote tumor metastasis independent of VEGF-C and lymphangiogenesis [68]. These TAMs were closely associated with existing lymphatic vessels through β4 integrin interactions with laminin-5. TGF-β1 produced by TAMs promoted TAM attachment to LECs and also reorganized LEC architecture to favor metastasis.

VEGF-C/VEGFR-3 signaling within TAMs also drives immune tolerance within the colorectal tumor microenvironment [69]. VEGFR-3 signaling was critical for the development of pro-inflammatory macrophages, which was associated with decreased presence of CD8 T cells within colorectal tumors. Blocking VEGFR-3 decreased the presence of TAMs, but increased their antigen processing and cross-presentation, leading to decreased tumor growth. 

## 7. Alternative Routes of Metastatic Dissemination

In addition to lymphatic transport, cancer cells at the primary tumor or distant site may take a different route to distant organs, primarily through blood vessels. It is unclear whether cancer cells preferentially disseminate from the primary site through blood or lymphatic vessels, but the frequency and site of metastatic spread is cancer-dependent [70]. Once in secondary organs, again cancer cells may further metastasize by lymphogenous or hematogenous route. Whole-exome sequencing of samples from 20 breast cancer patients suggested that nodal breast metastases did not seed distant metastasis. Instead, the authors conclude that distant organ metastases arise primarily from primary tumors, likely through a hematogenous route [71]. In another study, biopsied tissue of 17 colorectal cancer patients was sequenced to identify polyguanine repeats. The repeats allowed tracing of the evolutionary history of metastatic lymph nodes and liver metastases from the primary tumor [72]. This study found that 35% of distant metastases were seeded by metastatic lymph nodes, while 65% were seeded from the primary tumor, likely through a hematogenous route. These data show the heterogeneity in possible outcomes for cancer cells that arrive in the lymph node.

Once in lymph nodes, cancer cells can move along with lymph drainage or re-access the lymphatic system within the lymph node parenchyma [73] and may eventually enter the blood circulation through lymphovenous connections. Two recent studies suggest that cancer cells can directly enter the blood stream through blood vessels in lymph nodes. Once cancer cells exit nodes through blood vessels, they are able to colonize distant organs [14,15]. Immunohistochemical staining of human specimens show similar associations between cancer cells and blood vessels within metastatic nodes, but more work is needed to definitively track this route of dissemination in human cancer. Unlike targeting lymphangiogenesis in lung [22], inhibiting lymphangiogenesis in metastatic lymph nodes may not attenuate further metastatic spread.

## 8. Abnormal Tumor-Associated Lymphatic Vessels

### 8.1. Increased Lymphatic Permeability

Tumor-associated lymphatics are structurally and functionally abnormal, as are tumor blood vessels. The inability of lymphatic vessels to remove interstitial fluid from tumors is a contributing factor to the elevated interstitial fluid pressure and edema found in most tumors [74]. Although initial lymphatic vessels have a discontinuous basement membrane, tumor-associated lymphatic vessels become more permeable and less able to create and retain lymph (Figure 1). The discontinuities in lymphatic capillary walls not only allow transport of extracellular content both into and out of the vessel, but also allow entry by cancer cells, providing a route for metastatic dissemination. In addition to stimulating lymphangiogenesis, VEGF-C can render lymphatic vessels more permeable, as shown by dextran leakage from lymphatic vessels of mice transduced with adenoviral VEGF-C [75]. VEGF-C increased the permeability of intestinal lymphatic vessels, resulting in enhanced colorectal cancer metastasis [76]. Macrophages, which are also a source of VEGF-C, promote interendothelial gaps in LEC monolayers, providing an opportunity for enhanced cancer cell intravasation [67]. In addition, macrophages associated with LECs reduce the spread area of individual LECs, thus increasing lymphatic vessel permeability that may promote lymphatic metastasis [68]. Inflammation [77], chemotherapy [78], obesity [79], and tumor metabolites [73] have all been shown to increase lymphatic permeability. A recent study demonstrated that age can also affect lymphatic permeability. In older melanoma patients, cancer cells enter initial lymphatic capillaries but extravasate from collecting lymphatic vessels due to a loss of lymphatic vessel integrity. Consequently, melanoma cells avoid accumulation in tumor-draining lymph nodes but dissemination to distant sites is increased [80]. 

Few studies have sought to reverse lymphatic vessel permeability in vivo. Vascular cell adhesion molecule 1 (VCAM-1) is upregulated on LECs by tumor inflammation and was shown to regulate lymphatic vessel permeability [81]. Blocking VCAM-1 in vitro and in vivo reduced tumor-mediated lymphatic permeability and lymphatic invasion, suggesting reducing tumor-induced lymphatic vessel permeability may be a feasible approach to regulate lymphatic metastasis.

### 8.2. Altered Lymphatic Flow

Tumors increase both the number and size of surrounding tumor lymphatic vessels. VEGF-C and VEGF-D promote lymphangiogenesis and hyperplasia of small peritumoral lymphatic vessels, which increases the opportunity for cancer cells to enter lymphatic vessels. However, some nascent lymphatic vessels have been shown to be dysfunctional as they exhibit valve defects [82] and abnormal flow patterns. Although tumor inflammation can reduce contraction of tumor-draining collecting lymphatic vessels, lymphatic dilation due to VEGF-C and VEGF-D increases lymph flow and lymph node metastasis [82,83,84]. The impact of lymph drainage on remodeling the microenvironment and suppressing the host immune response have been described elsewhere [85]. Blocking VEGFR-2 or VEGFR-3 signaling reduced collecting lymphatic vessel dilation, which was associated with decreased lymph flow and nodal metastasis [84]. However, anti-VEGFR-3 treatment did not correct the multidirectional lymph flow pattern in peritumoral lymphatic vessels [82] and it is unclear whether immunosuppression is reversible after attenuating lymph/interstitial flow.

Transgenic mice with lymphatic insufficiency show that lymphatics are critical for drainage of extracellular fluid to prevent tumor edema [8,86,87]. Peritumoral edematous fluid contains high concentrations of cytokines that recruit leukocytes, immune-suppressive regulatory T cells, and myeloid derived suppressor cells [86]. Lymphatic vessels likely provide an exit for immune cells during inflammation, although lymphatics may also be important for initiating and modulating inflammation [86,87].

## 9. Immunomodulation by Lymphatics

Effector T cells home to tumors via blood vessels. Abnormal blood vessels caused by excessive VEGF-A and other pro-angiogenic factors interfere with T cell trafficking [88]. Recently, anti-angiogenesis therapy has been used to reverse the immunosuppressive microenvironment of tumors [89]. A challenge for anti-lymphangiogenic therapy is uncoupling tumor-associated lymphatic vessels from their roles in lymphatic metastasis and immune modulation. Lymphatic vessels are critical for the transport of tumor antigens by dendritic cells (DCs) to initiate anti-tumor immunity in lymph nodes and thus mice lacking dermal lymphatic vessels fail to mount adaptive immune responses against tumors [87]. 

Moreover, lymphangiogenesis can enhance the efficacy of cancer immunotherapy. High serum levels of VEGF-C are indicative of melanoma patients’ responses to immunotherapy, suggesting that elevated VEGF-C levels in circulation may be a biomarker for immunotherapy, despite its association with lymphatic metastasis [90]. Mechanistically, tumor-associated lymphatic vessels recruit T cells into melanoma tumors through CCL21. VEGF-C/VEGFR-3 signaling increased the number of activated T cells within primary melanoma lesions. Similarly, meningeal lymphangiogenesis induced by VEGF-C resulted in an enhanced immune response against glioblastoma tumors [91]. This response was more profoundly beneficial in combination with immune checkpoint inhibitors and VEGF-C. Thus, anti-lymphangiogenesis therapy may be incompatible with immune checkpoint blockade.

In contrast to the beneficial effects on immune surveillance, lymph from solid tumors delivers immunosuppressive molecules to attenuate anti-tumor immunity [92]. Antigen transported by lymphatic vessels can be presented by LECs, which use it to tolerize T cells [93]. Moreover, LECs actively dampen T cell mediated responses through PD-L1 expression [94]. The differential effects of lymphatic vessels on anti-tumor immunity must be considered with the use of anti-lymphangiogenic therapy.

## 10. Summary and Future Directions

Modulating lymphatic vessels to reduce metastatic spread has great therapeutic potential. However, the multiple functions of lymphatic vessels must be considered for targeting the process of lymphangiogenesis associated with cancer. In additional to the role of lymphatic vessels in fluid and cancer transport, evidence is emerging on how lymphatic vessels regulate innate and adaptive immunity, which may affect cancer progression. While blocking VEGFR-3 has been thought to not have an effect on existing lymphatic vessels, it was shown that VEGFR-3 is necessary for the maintenance of adult meningeal lymphatics [95]. An ongoing challenge is to identify pathways that decrease cancer cell recruitment and access to lymphatic vessels while preserving the physiological function of lymphatic vessels. Identifying and targeting pathways to normalize, or restore, tumor-associated lymphatic vessels may represent a viable therapeutic strategy. Finally, more work needs to be done to study the effects of blocking lymphangiogenesis in metastatic settings in order to address whether anti-lymphangiogenic drugs will be beneficial to patients with established metastases.
